# Tourism trends in the world׳s main destinations before and after the 2008 financial crisis using UNWTO official data

**DOI:** 10.1016/j.dib.2016.03.043

**Published:** 2016-04-01

**Authors:** Oscar Claveria, Alessio Poluzzi

**Affiliations:** AQR-IREA, University of Barcelona; International Tourism Destinations Consultant, Spain

**Keywords:** Tourism trends, Tourist destinations, UNWTO data

## Abstract

The first decade of the present century has been characterized by several economic shocks such as the 2008 financial crisis. In this data article we present the annual percentage growth rates of the main tourism indicators in the world׳s top tourist destinations: the United States, China, France, Spain, Italy, United Kingdom, Germany, Turkey, Mexico and Austria. We use data from the Compendium of Tourism Statistics provided by the World Tourism Organization (http://www2.unwto.org/content/data-0). It has been demonstrated that the dynamics of growth in the tourism industry pose different challenges to each destination in the previous study “Positioning and clustering of the world׳s top tourist destinations by means of dimensionality reduction techniques for categorical data” (Claveria and Poluzzi, 2016, [1]). We provide a descriptive analysis of the variables over the period comprised between 2000 and 2010. We complement the analysis by graphing the evolution of the main variables so as to visually represent the co-movements between tourism variables and economic growth.

**Specifications Table**

**Table:** 

Subject area	Economics, Tourism
More specific subject area	Destination marketing
Type of data	Tables, graphs
How data was acquired	Data from the *Compendium of Tourism Statistics* provided by the UNWTO and the World Bank.
Data format	Raw
Experimental factors	We have calculated the annual percentage growth rates of the series in levels (raw data)
Experimental features	We capture the dynamics of interaction between tourism indicators and economic growth in the world׳s top tourist destinations
Data source location	Top ten world destinations: the United States, China, France, Spain, Italy, United Kingdom, Germany, Turkey, Mexico and Austria
Data accessibility	Data is within this article

## 1 Value of the data

• The data presented in this article provide an overview of the main trends in the world׳s top ten destinations during the years preceding and after the 2008 financial crisis.
• The graphical representation of the series offers an outlook of the dynamic of interactions between the main tourism indicators and economic growth.
• This dataset can be used in destination positioning studies based on official macro data.

## 2 Data

The data included in this article were obtained from the Compendium of Tourism Statistics of the UNWTO (http://www2.unwto.org/content/data-0). We also use information about the evolution of GDP retrieved from the World Bank web (http://data.worldbank.org/indicator/NY.GDP.MKTP.KD.ZG). The main indicators provided by the UNWTO include: overnight visitors (thousands), total expenditure (US$ millions), occupancy rate (%), rooms, and inbound expenditure per GDP (%). ‘Overnight visitors’ stands for inbound tourism.

## 3 Experimental design, materials and methods

The omission of economic indicators and the lack of attention paid to economic return in tourism studies [2], has led us to incorporate economic information. Given that ratios provide insight into the profitability and the sustainability of tourism activities, we have calculated the ratio of expenditure per tourist as a proxy of tourism profitability. Annual percentage growth rates of Gross Domestic Product (GDP) and of total inbound expenditure over GDP were also calculated. By using annual percentage growth rates instead of levels, we avoided the issues derived from working with non-stationary time series.

In this work we present a descriptive analysis of the annual percentage growth rates of the main indicators provided by the UNWTO and the GDP at market prices based on constant local currency provided by the World Bank. Given that annual percentage growth rates are dimensionless measures of the amount of increase (or decrease) of a specific variable from one year to another in percentage terms, the evolution of the different tourism indicators and economic growth can be compared (Table 1, Table 2, Table 3, Table 4, Table 5, Table 6, Table 7).

**Fig. 1:**
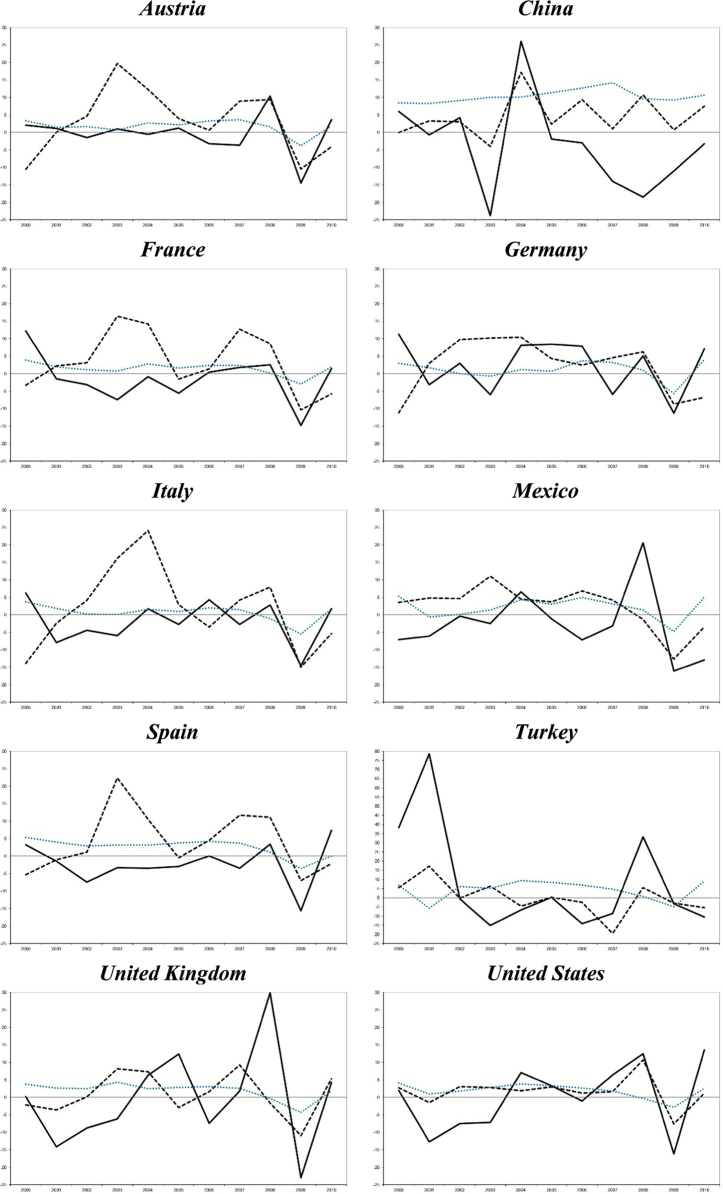
Inbound expenditure with respect to GDP vs. expenditure per tourist.

**Fig. 2:**
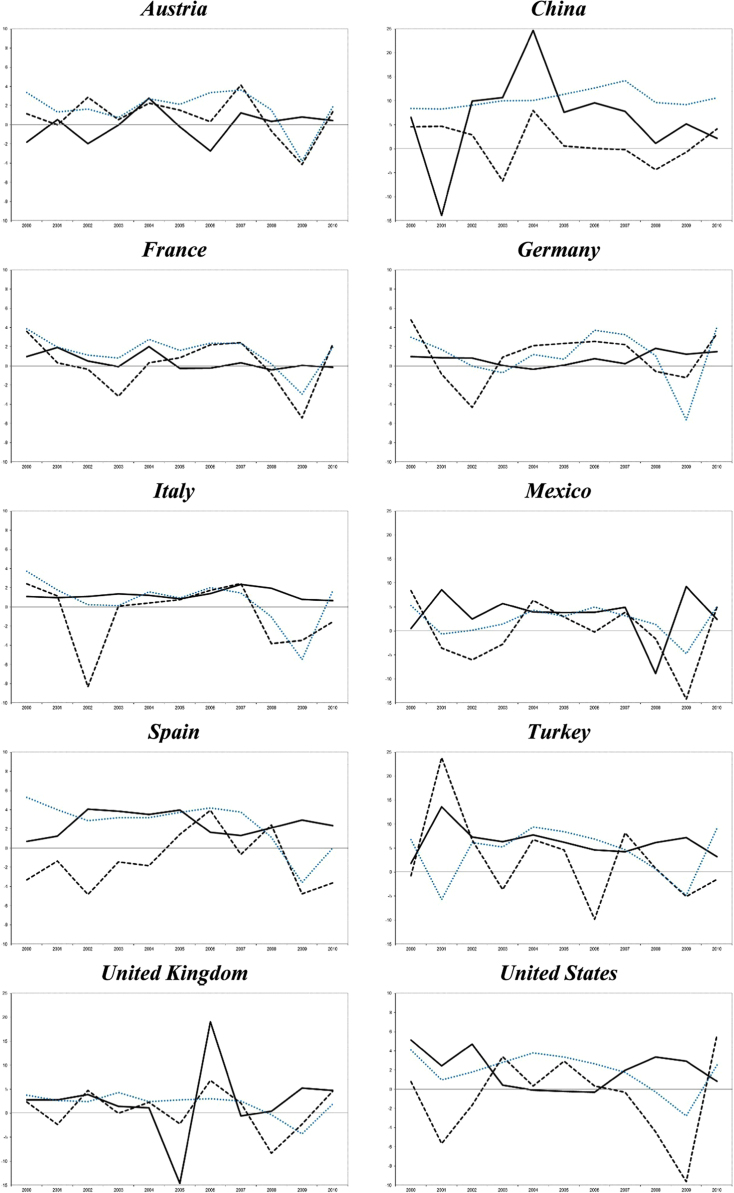
Total rooms vs. occupancy in each country.

In order to visually represent the evolution the interactions between the main tourism indicators and the economic growth in each destination, we complete the descriptive analysis with a graphical analysis of the annual percentage growth rates of the main variables (Figs. 1 and 2).

## Figures and Tables

**Table 1:** Annual percentage growth rates of international overnight visitors – (2000–2010).

Country	Mean	Median	Standard Deviation	Maximum	Minimum	Skewness	Kurtosis
Austria	2.14	2.49	1.98	5.59	−2.64	−0.92	4.83
China	7.23	9.41	10.20	26.66	−10.41	0.01	2.77
France	0.59	0.75	3.02	5.53	−3.10	0.24	1.66
Germany	4.33	3.61	5.70	10.96	−5.91	−0.35	1.97
Italy	1.80	0.60	6.23	12.78	−6.40	0.77	2.46
Mexico	1.98	1.18	5.38	10.46	−5.09	0.18	1.61
Spain	1.43	2.12	4.14	6.65	−8.77	−1.36	4.50
Turkey	15.61	14.05	14.76	39.07	−6.69	0.28	2.08
UK	1.98	0.70	6.89	12.69	−9.61	−0.06	2.08
US	2.16	3.60	7.36	11.81	−8.41	−0.27	1.53

Note: The Skewness and the Kurtosis indicators respectively measure the asymmetry and the shape (“peakedness”) of the probability distribution. Negative skew indicates that the tail on the left side of the probability density function is longer than the right side.

**Table 2:** Annual percentage growth rates of total expenditure – (2000–2010).

Country	Mean	Median	Standard Deviation	Maximum	Minimum	Skewness	Kurtosis
Austria	5.41	7.07	10.48	22.65	−12.84	−0.16	2.28
China	12.51	14.40	15.29	48.37	−13.96	0.69	4.45
France	4.01	5.34	8.77	16.99	−13.16	−0.32	2.55
Germany	6.56	8.33	8.95	20.88	−11.11	−0.50	2.67
Italy	3.20	4.74	9.50	16.20	−13.99	−0.23	2.12
Mexico	4.37	4.77	7.86	15.42	−14.83	−1.11	4.50
Spain	5.66	6.14	10.15	23.67	−15.18	−0.33	3.22
Turkey	15.28	18.22	15.99	46.76	−9.04	0.31	2.65
UK	3.14	6.04	11.13	20.92	−16.68	−0.42	2.38
US	4.03	8.47	9.65	14.56	−12.32	−0.56	1.81

Note: See Note of Table 1.

**Table 3:** Annual percentage growth rates of the occupancy rate – (2000–2010).

Country	Mean	Median	Standard Deviation	Maximum	Minimum	Skewness	Kurtosis
Austria	0.86	1.16	2.14	4.15	−4.14	−0.87	4.00
China	1.19	0.56	4.25	7.98	−6.67	−0.33	2.45
France	0.21	0.34	2.63	3.61	−5.41	−0.86	3.02
Germany	1.03	2.09	2.57	4.79	−4.32	−0.62	2.79
Italy	−0.74	0.43	3.31	2.45	−8.33	−1.15	3.42
Mexico	−0.15	−0.19	6.49	8.41	−14.18	−0.72	3.01
Spain	−1.27	−1.45	2.88	3.95	−4.82	0.45	2.11
Turkey	2.74	0.76	8.97	23.90	−9.77	1.01	3.94
UK	0.71	2.13	4.31	6.82	−8.33	−0.60	2.83
US	−0.75	0.32	4.41	5.69	−9.62	−0.58	2.67

Note: See Note of Table 1.

**Table 4:** Annual percentage growth rates of the number of rooms – (2000–2010).

Country	Mean	Median	Standard Deviation	Maximum	Minimum	Skewness	Kurtosis
Austria	−0.05	0.35	1.59	2.79	−2.72	−0.14	2.47
China	6.49	7.61	9.16	24.68	−13.91	−0.35	4.46
France	0.43	0.06	0.85	2.01	−0.40	0.99	2.54
Germany	0.73	0.83	0.66	1.84	−0.34	0.03	2.09
Italy	1.25	1.10	0.50	2.34	0.67	1.05	3.18
Mexico	3.33	3.88	4.81	9.30	−8.90	−1.39	5.04
Spain	2.52	2.34	1.21	4.06	0.69	−0.03	1.55
Turkey	6.22	6.24	3.07	13.63	1.84	1.05	4.34
UK	2.40	2.77	7.74	19.06	−14.64	−0.07	4.95
US	1.93	1.98	1.96	5.13	−0.30	0.34	1.78

Note: See Note of Table 1.

**Table 5:** Annual percentage growth rates of inbound expenditure per GDP – (2000–2010).

Country	Mean	Median	Standard Deviation	Maximum	Minimum	Skewness	Kurtosis
Austria	−0.37	0.94	6.02	10.35	−14.48	−0.74	4.46
China	−3.61	−2.98	13.54	26.04	−23.77	0.61	3.31
France	−1.36	−0.89	6.76	12.17	−14.77	−0.04	3.57
Germany	2.23	5.09	7.51	11.30	−11.30	−0.55	1.88
Italy	−1.95	−2.74	6.12	6.28	−14.55	−0.59	2.65
Mexico	−2.65	−3.13	9.84	20.58	−16.05	1.06	4.02
Spain	−2.16	−3.01	6.08	7.34	−15.63	−0.65	3.50
Turkey	8.31	−3.24	29.40	78.57	−15.07	1.43	3.89
UK	−0.42	0.22	14.17	29.89	−23.05	0.56	3.22
US	0.01	1.95	9.88	13.53	−16.19	−0.22	1.88

Note: See Note of Table 1.

**Table 6:** Annual percentage growth rates of total expenditure per tourist – (2000–2010).

Country	Mean	Median	Standard Deviation	Maximum	Minimum	Skewness	Kurtosis
Austria	3.14	3.97	9.33	19.65	−10.54	0.03	2.26
China	4.66	3.09	5.97	17.13	−3.96	0.70	2.81
France	3.44	2.25	8.66	16.42	−10.39	0.09	1.89
Germany	2.21	4.32	7.70	10.45	−11.20	−0.65	2.03
Italy	1.76	2.88	11.75	24.14	−14.99	0.35	2.53
Mexico	2.37	4.21	6.23	11.01	−12.60	−1.22	4.21
Spain	4.14	1.17	8.90	22.40	−7.03	0.65	2.55
Turkey	−0.04	−0.33	9.16	17.20	−19.40	−0.25	3.66
UK	0.97	0.11	6.12	9.25	−10.94	−0.28	2.38
US	1.74	1.90	4.28	10.68	−7.57	−0.16	4.60

Note: See Note of Table 1.

**Table 7:** Annual percentage growth rates of GDP at market prices – (2000–2010).

Country	Mean	Median	Standard Deviation	Maximum	Minimum	Skewness	Kurtosis
Austria	1.69	1.88	3.62	−3.80	2.04	−1.83	5.93
China	10.33	10.02	14.19	8.30	1.81	0.94	2.96
France	1.47	1.95	3.88	−2.94	1.77	−1.33	4.71
Germany	1.12	1.18	4.09	−5.64	2.73	−1.33	4.48
Italy	0.64	1.47	3.71	−5.48	2.37	−1.57	5.28
Mexico	2.14	3.03	5.30	−4.70	3.05	−0.95	3.23
Spain	2.52	3.19	5.29	−3.57	2.49	−1.44	4.30
Turkey	4.26	6.16	9.36	−5.70	5.29	−1.00	2.59
UK	1.94	2.56	4.30	−4.31	2.38	−1.82	5.49
US	1.88	2.53	4.09	−2.78	2.00	−1.18	3.72

Note: See Note of Table 1.
