# Supplementary material for: Tourism trends in the world׳s main destinations before and after the 2008 financial crisis using UNWTO official data
Source: Data Brief. 2016 Apr 1;7:1063–9. doi: 10.1016/j.dib.2016.03.043 (PMC5063797; doi:10.1016/j.dib.2016.03.043)
Supplement: Supplementary file 1 — Supplementary material [file mmc1.pdf]

CONFLICT OF INTEREST FORM

It is important that you return this form upon submission. We will not publish your article without completion and return of this form.

Title of Paper: Tourism trends in the world's main destinations before and after the 2008 financial crisis using UNWTO data

Please tick one of the following boxes:

☒ We have no conflict of interest to declare.

☐ We have a competing interest to declare (please fill in box below):

This statement is to certify that all Authors have seen and approved the manuscript being submitted. We warrant that the article is the Authors' original work. We warrant that the article has not received prior publication and is not under consideration for publication elsewhere. On behalf of all Co-Authors, the corresponding Author shall bear full responsibility for the submission.

**Author Signature**	**Print Name**
	Oscar Claveria

☒ Please check this box if you are submitting this on behalf of all authors.
