# Supplementary material for: Tourism trends in the world׳s main destinations before and after the 2008 financial crisis using UNWTO official data
Source: Data Brief. 2016 Apr 1;7:1063–9. doi: 10.1016/j.dib.2016.03.043 (PMC5063797; doi:10.1016/j.dib.2016.03.043)
Supplement: Supplementary file 2 — Supplementary material [file mmc2.zip › Tables and figures_dib_R2.docx]

**Table 1**

Annual percentage growth rates of international overnight visitors – (2000-2010)

| Country | Mean | Median | Standard  Deviation | Maximum | Minimum | Skewness | Kurtosis |
| --- | --- | --- | --- | --- | --- | --- | --- |
| Austria | 2.14 | 2.49 | 1.98 | 5.59 | -2.64 | -0.92 | 4.83 |
| China | 7.23 | 9.41 | 10.20 | 26.66 | -10.41 | 0.01 | 2.77 |
| France | 0.59 | 0.75 | 3.02 | 5.53 | -3.10 | 0.24 | 1.66 |
| Germany | 4.33 | 3.61 | 5.70 | 10.96 | -5.91 | -0.35 | 1.97 |
| Italy | 1.80 | 0.60 | 6.23 | 12.78 | -6.40 | 0.77 | 2.46 |
| Mexico | 1.98 | 1.18 | 5.38 | 10.46 | -5.09 | 0.18 | 1.61 |
| Spain | 1.43 | 2.12 | 4.14 | 6.65 | -8.77 | -1.36 | 4.50 |
| Turkey | 15.61 | 14.05 | 14.76 | 39.07 | -6.69 | 0.28 | 2.08 |
| UK | 1.98 | 0.70 | 6.89 | 12.69 | -9.61 | -0.06 | 2.08 |
| US | 2.16 | 3.60 | 7.36 | 11.81 | -8.41 | -0.27 | 1.53 |

Note: The Skewness and the Kurtosis indicators respectively measure the asymmetry and the shape (“peakedness”) of the probability distribution. Negative skew indicates that the tail on the left side of the probability density function is [longer](https://en.wikipedia.org/wiki/Long_tail) than the right side.

**Table 2**

Annual percentage growth rates of total expenditure – (2000-2010)

| Country | Mean | Median | Standard  Deviation | Maximum | Minimum | Skewness | Kurtosis |
| --- | --- | --- | --- | --- | --- | --- | --- |
| Austria | 5.41 | 7.07 | 10.48 | 22.65 | -12.84 | -0.16 | 2.28 |
| China | 12.51 | 14.40 | 15.29 | 48.37 | -13.96 | 0.69 | 4.45 |
| France | 4.01 | 5.34 | 8.77 | 16.99 | -13.16 | -0.32 | 2.55 |
| Germany | 6.56 | 8.33 | 8.95 | 20.88 | -11.11 | -0.50 | 2.67 |
| Italy | 3.20 | 4.74 | 9.50 | 16.20 | -13.99 | -0.23 | 2.12 |
| Mexico | 4.37 | 4.77 | 7.86 | 15.42 | -14.83 | -1.11 | 4.50 |
| Spain | 5.66 | 6.14 | 10.15 | 23.67 | -15.18 | -0.33 | 3.22 |
| Turkey | 15.28 | 18.22 | 15.99 | 46.76 | -9.04 | 0.31 | 2.65 |
| UK | 3.14 | 6.04 | 11.13 | 20.92 | -16.68 | -0.42 | 2.38 |
| US | 4.03 | 8.47 | 9.65 | 14.56 | -12.32 | -0.56 | 1.81 |

Note: See Note of Table 1.

**Table 3**

Annual percentage growth rates of the occupancy rate – (2000-2010)

| Country | Mean | Median | Standard  Deviation | Maximum | Minimum | Skewness | Kurtosis |
| --- | --- | --- | --- | --- | --- | --- | --- |
| Austria | 0.86 | 1.16 | 2.14 | 4.15 | -4.14 | -0.87 | 4.00 |
| China | 1.19 | 0.56 | 4.25 | 7.98 | -6.67 | -0.33 | 2.45 |
| France | 0.21 | 0.34 | 2.63 | 3.61 | -5.41 | -0.86 | 3.02 |
| Germany | 1.03 | 2.09 | 2.57 | 4.79 | -4.32 | -0.62 | 2.79 |
| Italy | -0.74 | 0.43 | 3.31 | 2.45 | -8.33 | -1.15 | 3.42 |
| Mexico | -0.15 | -0.19 | 6.49 | 8.41 | -14.18 | -0.72 | 3.01 |
| Spain | -1.27 | -1.45 | 2.88 | 3.95 | -4.82 | 0.45 | 2.11 |
| Turkey | 2.74 | 0.76 | 8.97 | 23.90 | -9.77 | 1.01 | 3.94 |
| UK | 0.71 | 2.13 | 4.31 | 6.82 | -8.33 | -0.60 | 2.83 |
| US | -0.75 | 0.32 | 4.41 | 5.69 | -9.62 | -0.58 | 2.67 |

Note: See Note of Table 1.

**Table 4**

Annual percentage growth rates of the number of rooms – (2000-2010)

| Country | Mean | Median | Standard  Deviation | Maximum | Minimum | Skewness | Kurtosis |
| --- | --- | --- | --- | --- | --- | --- | --- |
| Austria | -0.05 | 0.35 | 1.59 | 2.79 | -2.72 | -0.14 | 2.47 |
| China | 6.49 | 7.61 | 9.16 | 24.68 | -13.91 | -0.35 | 4.46 |
| France | 0.43 | 0.06 | 0.85 | 2.01 | -0.40 | 0.99 | 2.54 |
| Germany | 0.73 | 0.83 | 0.66 | 1.84 | -0.34 | 0.03 | 2.09 |
| Italy | 1.25 | 1.10 | 0.50 | 2.34 | 0.67 | 1.05 | 3.18 |
| Mexico | 3.33 | 3.88 | 4.81 | 9.30 | -8.90 | -1.39 | 5.04 |
| Spain | 2.52 | 2.34 | 1.21 | 4.06 | 0.69 | -0.03 | 1.55 |
| Turkey | 6.22 | 6.24 | 3.07 | 13.63 | 1.84 | 1.05 | 4.34 |
| UK | 2.40 | 2.77 | 7.74 | 19.06 | -14.64 | -0.07 | 4.95 |
| US | 1.93 | 1.98 | 1.96 | 5.13 | -0.30 | 0.34 | 1.78 |

Note: See Note of Table 1.

**Table 5**

Annual percentage growth rates of inbound expenditure per GDP – (2000-2010)

| Country | Mean | Median | Standard  Deviation | Maximum | Minimum | Skewness | Kurtosis |
| --- | --- | --- | --- | --- | --- | --- | --- |
| Austria | -0.37 | 0.94 | 6.02 | 10.35 | -14.48 | -0.74 | 4.46 |
| China | -3.61 | -2.98 | 13.54 | 26.04 | -23.77 | 0.61 | 3.31 |
| France | -1.36 | -0.89 | 6.76 | 12.17 | -14.77 | -0.04 | 3.57 |
| Germany | 2.23 | 5.09 | 7.51 | 11.30 | -11.30 | -0.55 | 1.88 |
| Italy | -1.95 | -2.74 | 6.12 | 6.28 | -14.55 | -0.59 | 2.65 |
| Mexico | -2.65 | -3.13 | 9.84 | 20.58 | -16.05 | 1.06 | 4.02 |
| Spain | -2.16 | -3.01 | 6.08 | 7.34 | -15.63 | -0.65 | 3.50 |
| Turkey | 8.31 | -3.24 | 29.40 | 78.57 | -15.07 | 1.43 | 3.89 |
| UK | -0.42 | 0.22 | 14.17 | 29.89 | -23.05 | 0.56 | 3.22 |
| US | 0.01 | 1.95 | 9.88 | 13.53 | -16.19 | -0.22 | 1.88 |

Note: See Note of Table 1.

**Table 6**

Annual percentage growth rates of total expenditure per tourist – (2000-2010)

| Country | Mean | Median | Standard  Deviation | Maximum | Minimum | Skewness | Kurtosis |
| --- | --- | --- | --- | --- | --- | --- | --- |
| Austria | 3.14 | 3.97 | 9.33 | 19.65 | -10.54 | 0.03 | 2.26 |
| China | 4.66 | 3.09 | 5.97 | 17.13 | -3.96 | 0.70 | 2.81 |
| France | 3.44 | 2.25 | 8.66 | 16.42 | -10.39 | 0.09 | 1.89 |
| Germany | 2.21 | 4.32 | 7.70 | 10.45 | -11.20 | -0.65 | 2.03 |
| Italy | 1.76 | 2.88 | 11.75 | 24.14 | -14.99 | 0.35 | 2.53 |
| Mexico | 2.37 | 4.21 | 6.23 | 11.01 | -12.60 | -1.22 | 4.21 |
| Spain | 4.14 | 1.17 | 8.90 | 22.40 | -7.03 | 0.65 | 2.55 |
| Turkey | -0.04 | -0.33 | 9.16 | 17.20 | -19.40 | -0.25 | 3.66 |
| UK | 0.97 | 0.11 | 6.12 | 9.25 | -10.94 | -0.28 | 2.38 |
| US | 1.74 | 1.90 | 4.28 | 10.68 | -7.57 | -0.16 | 4.60 |

Note: See Note of Table 1.

**Table 7**

Annual percentage growth rates of GDP at market prices– (2000-2010)

| Country | Mean | Median | Standard  Deviation | Maximum | Minimum | Skewness | Kurtosis |
| --- | --- | --- | --- | --- | --- | --- | --- |
| Austria | 1.69 | 1.88 | 3.62 | -3.80 | 2.04 | -1.83 | 5.93 |
| China | 10.33 | 10.02 | 14.19 | 8.30 | 1.81 | 0.94 | 2.96 |
| France | 1.47 | 1.95 | 3.88 | -2.94 | 1.77 | -1.33 | 4.71 |
| Germany | 1.12 | 1.18 | 4.09 | -5.64 | 2.73 | -1.33 | 4.48 |
| Italy | 0.64 | 1.47 | 3.71 | -5.48 | 2.37 | -1.57 | 5.28 |
| Mexico | 2.14 | 3.03 | 5.30 | -4.70 | 3.05 | -0.95 | 3.23 |
| Spain | 2.52 | 3.19 | 5.29 | -3.57 | 2.49 | -1.44 | 4.30 |
| Turkey | 4.26 | 6.16 | 9.36 | -5.70 | 5.29 | -1.00 | 2.59 |
| UK | 1.94 | 2.56 | 4.30 | -4.31 | 2.38 | -1.82 | 5.49 |
| US | 1.88 | 2.53 | 4.09 | -2.78 | 2.00 | -1.18 | 3.72 |

Note: See Note of Table 1.

**Fig. 1.** Inbound expenditure with respect to GDP vs. expenditure per tourist

| ***Austria*** | ***China*** |
| --- | --- |
| **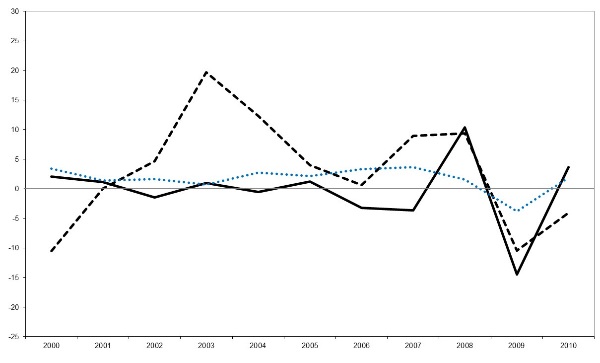** | **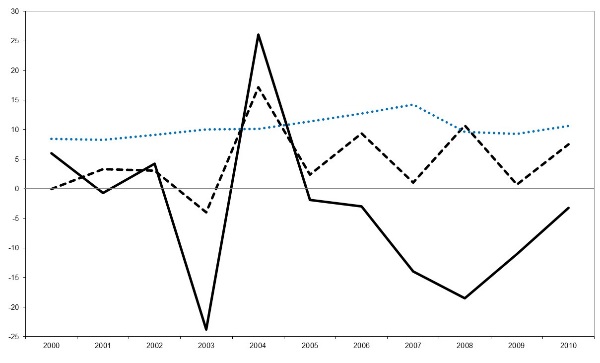** |
| ***France*** | ***Germany*** |
| **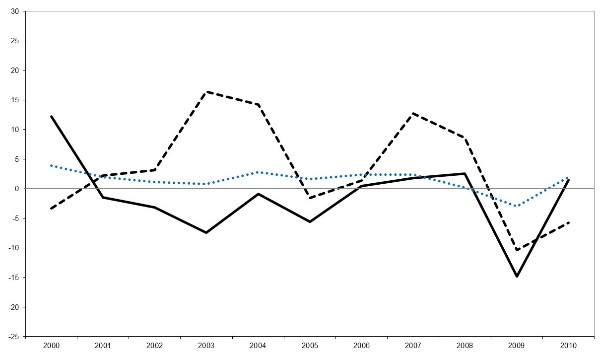** | **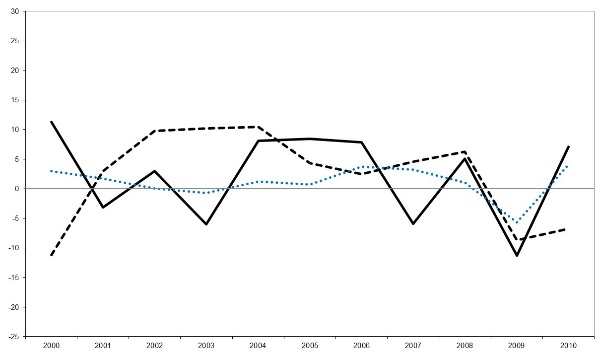** |
| ***Italy*** | ***Mexico*** |
| **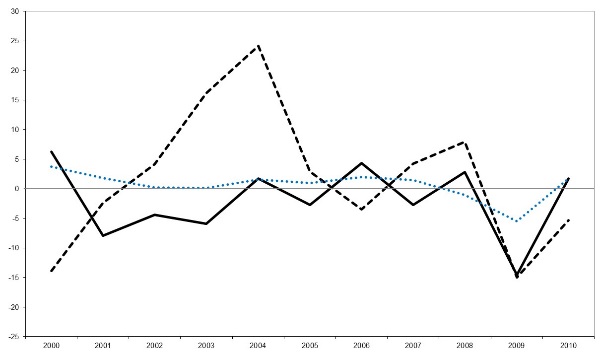** | **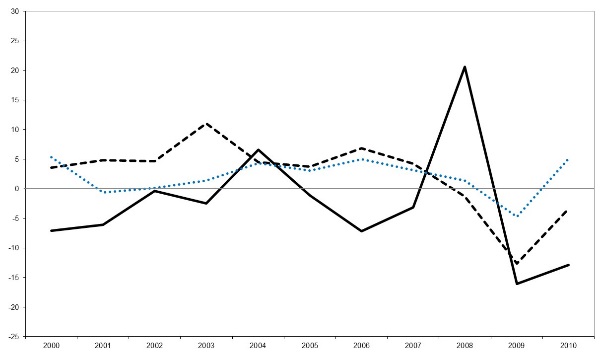** |
| ***Spain*** | ***Turkey*** |
| **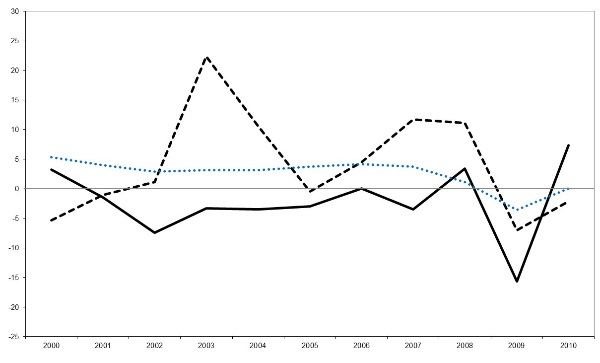** | **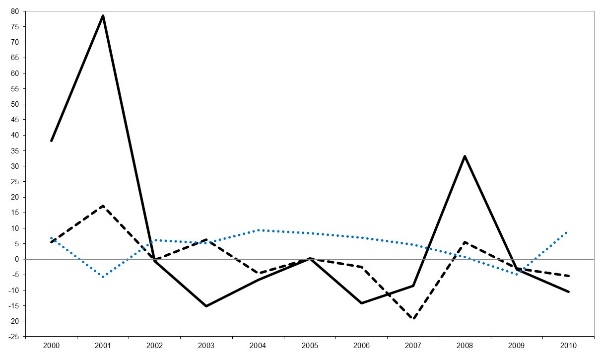** |
| ***United Kingdom*** | ***United States*** |
| **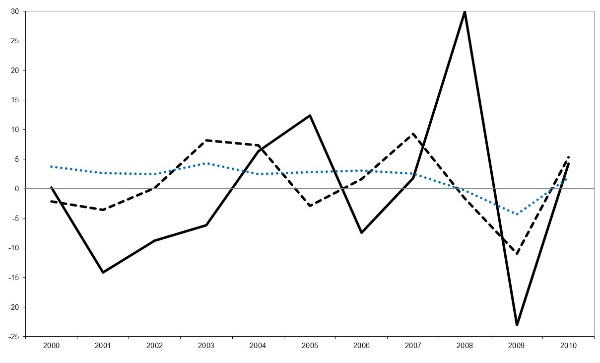** | **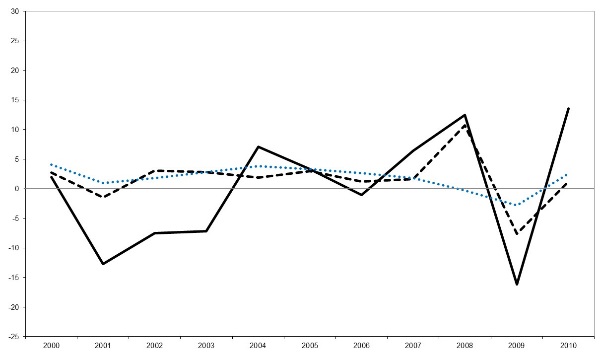** |

1. Note: Compiled by the author. The black line represents the annual growth rate of the inbound expenditure with respect to GDP in each country, and the black dotted line represents the growth rate of expenditure per tourist in each country. The grey dotted line represents the annual percentage growth rate of GDP in each country.

**Fig. 2.** Total rooms vs. occupancy in each country

| ***Austria*** | ***China*** |
| --- | --- |
| **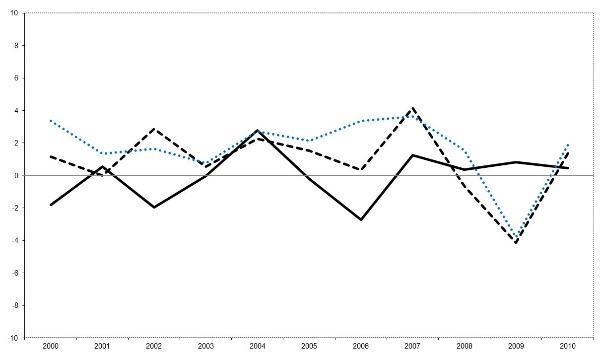** | **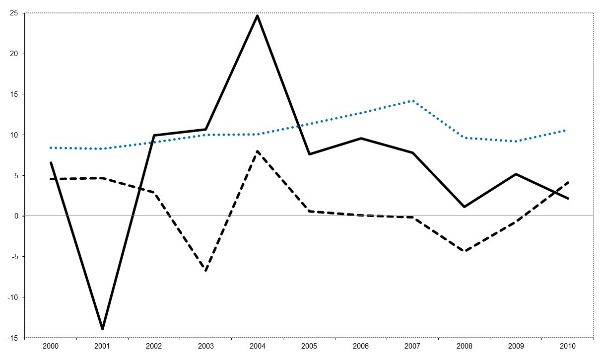** |
| ***France*** | ***Germany*** |
| **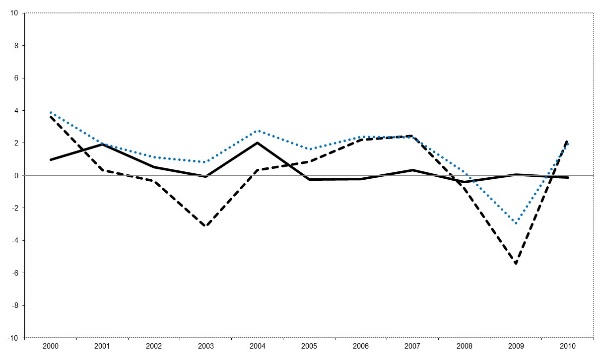** | **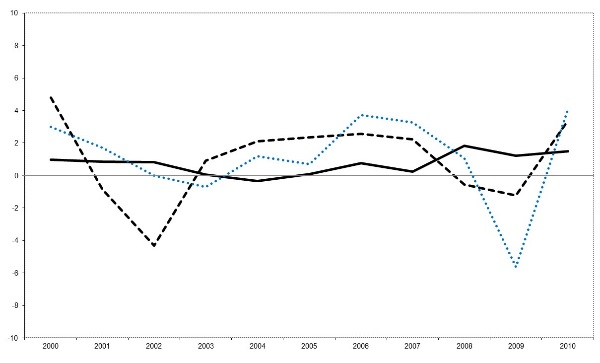** |
| ***Italy*** | ***Mexico*** |
| **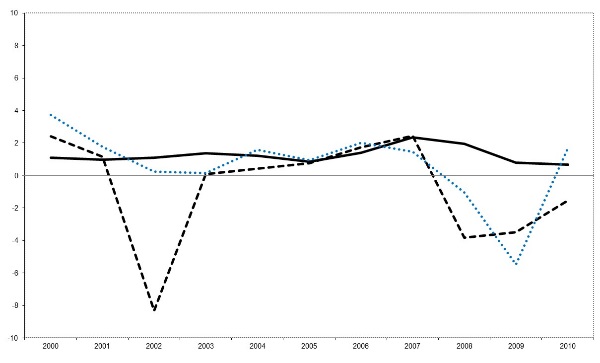** | **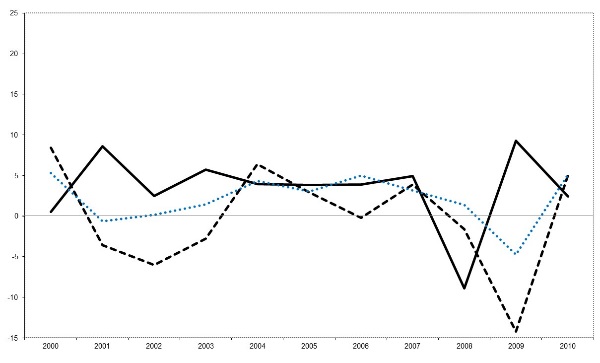** |
| ***Spain*** | ***Turkey*** |
| **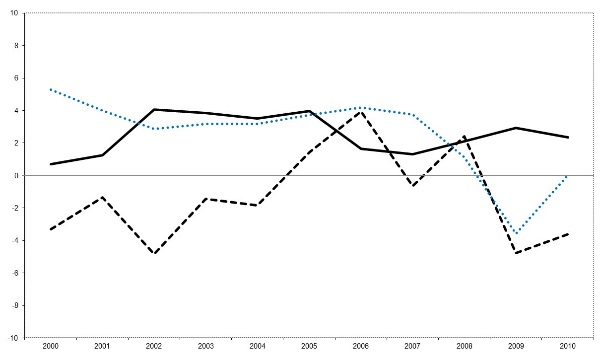** | **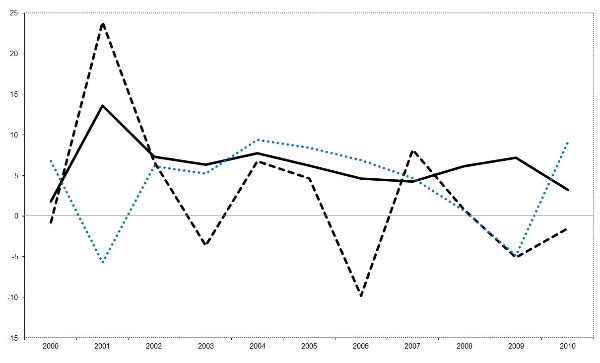** |
| ***United Kingdom*** | ***United States*** |
| **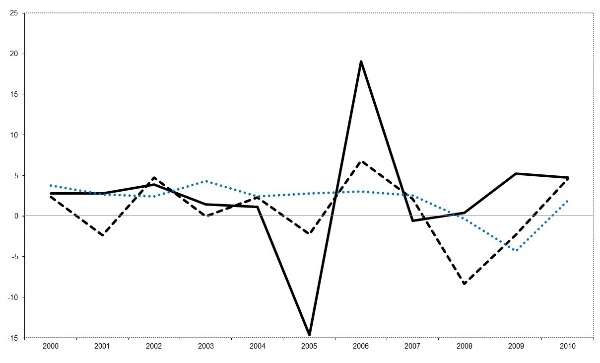** | **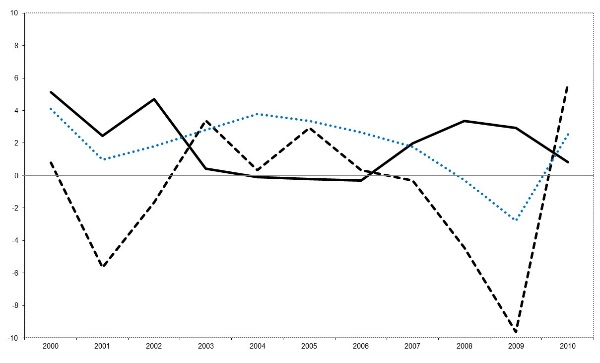** |

1. Note: Compiled by the author. The black line represents the annual growth rate of total rooms in each country, and the black dotted line represents the growth rate of total occupancy in each country. The grey dotted line represents the annual percentage growth rate of GDP in each country.
